# Associations of Preconception Exposure to Air Pollution and Greenness with Offspring Asthma and Hay Fever

**DOI:** 10.3390/ijerph17165828

**Published:** 2020-08-12

**Authors:** Ingrid Nordeide Kuiper, Iana Markevych, Simone Accordini, Randi J. Bertelsen, Lennart Bråbäck, Jesper Heile Christensen, Bertil Forsberg, Thomas Halvorsen, Joachim Heinrich, Ole Hertel, Gerard Hoek, Mathias Holm, Kees de Hoogh, Christer Janson, Andrei Malinovschi, Alessandro Marcon, Torben Sigsgaard, Cecilie Svanes, Ane Johannessen

**Affiliations:** 1 Department of Occupational Medicine, Haukeland University Hospital, 5020 Bergen, Norway; cecilie.svanes@helse-bergen.no; 2 Centre for International Health, Department of Global Public Health and Primary Care, University of Bergen, 5020 Bergen, Norway; ane.Johannessen@uib.no; 3 Institute and Clinic for Occupational, Social and Environmental Medicine, University Hospital, Ludwig Maximilian University of Munich, 85764 Munich, Germany; iana.markevych@uj.edu.pl (I.M.); joachim.Heinrich@med.uni-muenchen.de (J.H.); 4 Institute of Psychology, Jagiellonian University, 30-060 Krakow, Poland; 5 Institute of Epidemiology, Helmholtz Zentrum München-German Research Center for Environmental Health, 80336 Neuherberg, Germany; 6 Unit of Epidemiology and Medical Statistics, Department of Diagnostics and Public Health, University of Verona, 37134 Verona, Italy; simone.accordini@univr.it (S.A.); alessandro.marcon@univr.it (A.M.); 7 Department of Clinical Science, University of Bergen, 5020 Bergen, Norway; randi.J.Bertelsen@uib.no (R.J.B.); thomas.halvorsen@helse-bergen.no (T.H.); 8 Oral Health Centre of Expertise in Western Norway, 5020 Bergen, Norway; 9 Section of Sustainable Health, Department of Public Health and Clinical Medicine, Umea University, 901 87 Umea, Sweden; lennart.braback@umu.se (L.B.); bertil.forsberg@umu.se (B.F.); 10 Department of Environmental Science, Aarhus University, 4000 Roskilde, Denmark; jc@envs.au.dk (J.H.C.); oh@envs.au.dk (O.H.); 11 Allergy and Lung Health Unit, Melbourne School of Population and Global Health, University of Melbourne, Melbourne 3010, Australia; 12 Institute for Risk Assessment Sciences, Utrecht University, 3584 CS Utrecht, The Netherlands; g.hoek@uu.nl; 13 Department of Occupational and Environmental Medicine, University of Gothenburg, SE-405 30 Gothenburg, Sweden; mathias.holm@amm.gu.se; 14 Swiss Tropical and Public Health Institute, 4051 Basel, Switzerland; c.dehoogh@swisstph.ch; 15 University of Basel, 4001 Basel, Switzerland; 16 Department of Medical Sciences, Respiratory, Allergy & Sleep Research, Uppsala University, 751 85 Uppsala, Sweden; christer.janson@medsci.uu.se; 17 Department of Medical Sciences, Clinical Physiology, Uppsala University, 751 85 Uppsala, Sweden; andrei.malinovschi@medsci.uu.se; 18 Section of Environment, Occupation & Health, Institute of Public Health, Aarhus University, 8000 Aarhus, Denmark; ts@ph.au.dk

**Keywords:** air pollution, greenness, preconception exposure, childhood asthma, childhood hay fever

## Abstract

We investigated if greenness and air pollution exposure in parents’ childhood affect offspring asthma and hay fever, and if effects were mediated through parental asthma, pregnancy greenness/pollution exposure, and offspring exposure. We analysed 1106 parents with 1949 offspring (mean age 35 and 6) from the Respiratory Health in Northern Europe, Spain and Australia (RHINESSA) generation study. Mean particulate matter (PM_2.5_ and PM_10_), nitrogen dioxide (NO_2_), black carbon (BC), ozone (O_3_) (µg/m^3^) and greenness (normalized difference vegetation index (NDVI)) were calculated for parents 0–18 years old and offspring 0–10 years old, and were categorised in tertiles. We performed logistic regression and mediation analyses for two-pollutant models (clustered by family and centre, stratified by parental lines, and adjusted for grandparental asthma and education). Maternal medium PM_2.5_ and PM_10_ exposure was associated with higher offspring asthma risk (odds ratio (OR) 2.23, 95%CI 1.32–3.78, OR 2.27, 95%CI 1.36–3.80), and paternal high BC exposure with lower asthma risk (OR 0.31, 95%CI 0.11–0.87). Hay fever risk increased for offspring of fathers with medium O_3_ exposure (OR 4.15, 95%CI 1.28–13.50) and mothers with high PM_10_ exposure (OR 2.66, 95%CI 1.19–5.91). The effect of maternal PM_10_ exposure on offspring asthma was direct, while for hay fever, it was mediated through exposures in pregnancy and offspring’s own exposures. Paternal O_3_ exposure had a direct effect on offspring hay fever. To conclude, parental exposure to air pollution appears to influence the risk of asthma and allergies in future offspring.

## 1. Introduction

Air pollution is a major risk factor for disease worldwide and is estimated to cause almost 500,000 premature annual deaths across Europe [1]. Studies have shown that long-term exposure to high levels of air pollution affects multiple organs in the human body, causing cardiovascular and respiratory diseases [2]. Regarding the development of asthma, some studies have found childhood exposure to air pollution to be a risk factor [3], while other studies did not reveal those effects [4]. Less is known regarding the intergenerational effects of exposure to lower levels of air pollution, e.g., levels below recommended limits from the European Union (EU) and the World Health Organisation (WHO) [5,6], on offspring asthma and hay fever.

Exposure to greenspace has, on the other hand, been associated with beneficial health effects such as reduced risk of mortality, diabetes, and high blood pressure [7]. However, effects of greenness on asthma and allergies are less clear [8,9,10,11]. Some studies have indicated decreased respiratory morbidity in adulthood due to living near green areas [7,12,13,14] while the effects of residential greenness on childhood allergic rhinitis and aeroallergen sensitization have depended on the region [15,16,17,18]. Access to green areas may decrease stress through rest, increase opportunities for physical activity and increase social interaction [19]. Furthermore, vegetation may remove pollutants such as ozone (O_3_), particulate matter (PM) and nitrogen dioxide (NO_2_) from the air and may reduce exposure to harmful noise [9,20]. Negative effects of greenness, on the other hand, may be explained by higher exposure to pollen triggering allergic responses [17]. 

Asthma and allergies may result from both genetic susceptibility and environmental exposures, and the importance of early life factors have been widely acknowledged [21,22,23]. Emerging research suggests that even preconception exposures may be of relevance, and that epigenetic mechanisms may be at play across generations [24]. Recent studies have found that father’s smoking and overweight onset in adolescence was associated with higher asthma risk in their future offspring [25,26,27], suggesting vulnerable time windows many years before conception of offspring. There are, however, no studies investigating such intergenerational effects of exposures to air pollution and greenness. 

To address the knowledge gaps of these long-term effects of exposure to air pollution and greenness on asthma and hay fever, the aims of our study were to (1) explore the associations between parental childhood exposures of greenness and air pollution in relation to their future offspring asthma and allergies, in areas with relatively low air pollution and to (2) assess if the observed associations were direct or mediated by other factors.

## 2. Materials and Methods 

### 2.1. Study Design and Population

**Figure 1:**
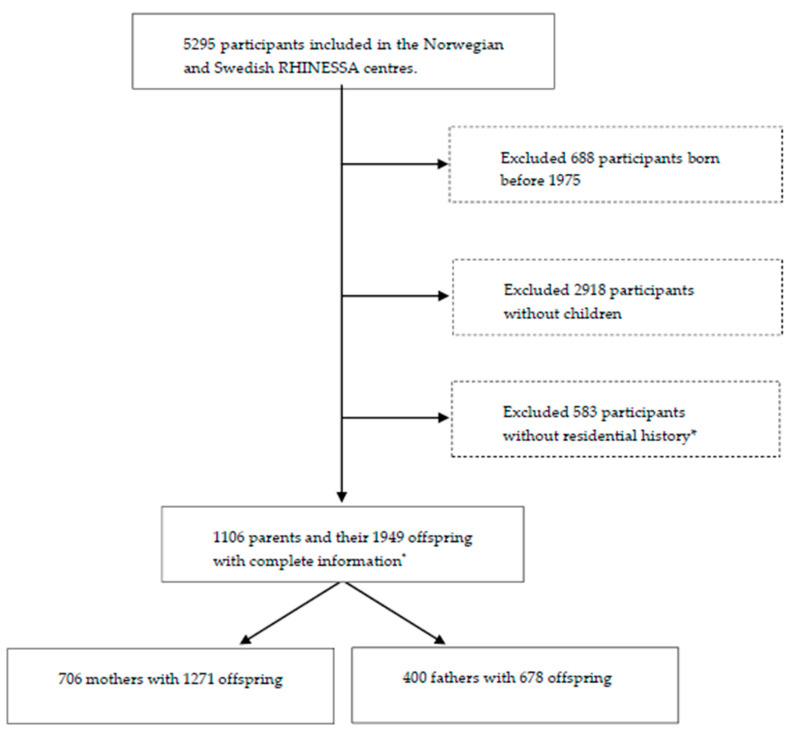
Flowchart of the Respiratory Health in Northern Europe, Spain and Australia (RHINESSA) generation study population. * Lack of residential history due to lack of registered addresses in the population registries or lack of consent to address history retrieval.

We included participants born after 1975 as well as their offspring from centres with available pollution data and relatively low air pollution levels in the Respiratory Health in Northern Europe, Spain and Australia (RHINESSA) generation study, conducted in 2013–2015 [28,29]: Bergen (Norway); and Umea, Uppsala, and Gothenburg (Sweden), as shown in Figure 1. Individual residential address history was only available from 1975 onwards and participants born before that were therefore not included. The participants answered questionnaires regarding their lung health and provided information on their offspring asthma and allergies. The overall response rate was 40% in Norway and 44% in Sweden [28]. Informed consent was obtained from each participant, and the study was approved by regional committees of medical research ethics according to national legislations [30].

### 2.2. Residential Address History

We retrieved the parents’ geocoded residential addresses from the Swedish and Norwegian national population registries for each year ranging from parents’ birth until the age of 18 years, as well as for offspring from birth until the age of 10 years. 

### 2.3. Outcomes

The main outcomes in this study were offspring early-onset asthma and hay fever, defined as affirmative answers to the questions “For each of your biological children, please tick yes if they have had asthma before 10 years of age”, and “For each of your biological children please tick yes if they have had hay fever/rhinitis”, respectively.

### 2.4. Exposure Assessment

#### 2.4.1. Air Pollution

We assigned annual mean concentrations (µg/m^3^) of 5 different air pollutants—NO_2_, PM_2.5_, PM_10_, black carbon (BC) and O_3_—to each participant based on their geocoded residential history. The exposures were assigned from air pollution rasters developed previously [31,32,33]. Annual mean PM_10_ exposures were extracted for 2005 to 2007 from surfaces (100 × 100 m) based on western Europe-wide hybrid land use regression (LUR) models [31]. Annual mean NO_2_, PM_2.5_ and O_3_ exposures and BC exposures for 2010 originate from similar hybrid LUR models [32,33]. An overview of the models used for the different pollutants can be found in the online supplement (Table S1).

We back-and-forth extrapolated the air pollution concentrations from the LUR models using the ratio method for each year from 1990 to 2015 following the procedure from the European Study of Cohorts for Air Pollution Effects (ESCAPE) project [34], that is based on the Danish Eulerian Hemispheric (DEHM) model [35]. For the years before 1990, we used 1990 estimates as proxies.

#### 2.4.2. Greenness

Greenness was assessed using the normalized difference vegetation index (NDVI) [36], which refers to both structured and unstructured vegetation. NDVI estimates were derived from cloud free Landsat 4–5 TM and 8 OLI satellite images [37] (Table S2). NDVI values range from −1 to +1, with +1 indicating highly vegetated areas [38].

Satellite images were retrieved for every 5 years during the most vegetation rich months (May, June, July) (Table S3), and NDVI maps were calculated with mean NDVI in a circular 100 m, 300 m, 500 m and 1000 m buffer around each participant’s residential address. In the main analysis, we included the 300 m buffer, while the other buffer zones were included in sensitivity analysis (Tables S4 and S6a,b).

### 2.5. Time Windows for Exposures

We averaged mean annual exposures for the air pollutants and greenness across the period 0–18 years of age for parents and 0–10 years of age for offspring. Although desirable to estimate separate exposures for parents’ childhood and adolescence, stable residential patterns made this unfeasible (Table S7a–f). 

### 2.6. Covariates and Mediators

To identify the minimal sufficient covariate adjustment set, we used a directed acyclic graph (DAG) (Figures S1–S4) [39,40]. To be considered as a confounder variable, the covariate had to be associated with both the exposure and the outcome and precede them both in time. Based on the DAG, we adjusted the multivariable analyses for grandparental education and grandparental asthma. Grandparental asthma was defined based on positive report by the parents on the question: “Have your biological parents ever had asthma?” with separate answer categories for “mother” and “father”. Grandparental education level was defined based on the question: “What was the highest level of education your mother/father has/had?”, with categories primary school, secondary school and college/university.

In addition, parental asthma, offspring’s own pollution/greenness exposures and pollution/greenness exposures during pregnancy (defined as birth year and the preceding year) were included as potential mediators based on a priori hypothesis that they may lie in the pathway between parental air pollution/greenness exposures and offspring asthma/allergies.

### 2.7. Statistical Analyses

All statistical analyses were performed using Stata version 16.0.

Descriptive analyses were stratified by parental sex.

We performed multilevel logistic regression analyses to investigate associations between air pollutants and greenness categorized in tertiles (low, medium and high exposures; see definition for all categories in Table S5), and early-onset asthma and hay fever as binary outcomes. The analyses were complete case analyses, clustered by family (to account for siblings) and study centre, and stratified by parental sex. All models were adjusted for O_3_ and NDVI (300 m buffer), except for the O_3_ model which was adjusted for NO_2_ and NDVI (300 m buffer) and the NDVI model which was adjusted for O_3_ and NO_2_. All models were also adjusted for grandparental education and grandparental asthma.

As sensitivity analyses, we fitted regression models separately for each country (Table S8a,b) and for parents born after 1985 (Table S9a,b). *p*-values < 0.05 were considered statistically significant.

Correlation analyses were performed for all exposures to decide which pollutants to include in the same models (Tables S10a,b and S11a–f).

**Figure 2:**
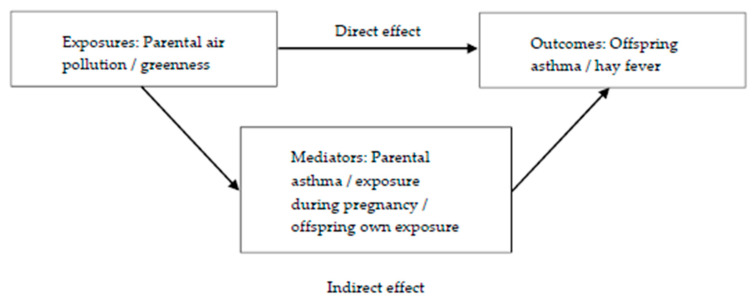
Mediation models for the effects of parental exposures (air pollution/greenness) on offspring’s outcomes (asthma or hay fever).

Mediation analyses were performed to decompose the total effects of parental exposures to greenness and each air pollutant on offspring’s outcomes into their direct and indirect (mediated) effects (Figure 2). Parental asthma, offspring’s exposure during pregnancy and offspring’s own exposure were all evaluated as potential mediators. In order to be a mediator, the exposure must be associated with the mediator and the mediator must be associated with the outcome. Mediation tests showed that offspring’s own exposure and exposure during pregnancy were potential mediators between maternal pollution exposure (PM_10_) and both offspring’s outcomes. For the paternal line, exposure during pregnancy (O_3_) was a potential mediator between paternal O_3_ exposure and offspring hay fever.

The mediation analyses were conducted using ldecomp in Stata, a simple counterfactual mediation method that requires a categorical main exposure variable and a binary outcome, and allows any distribution of the mediator [41,42]. We used bootstrapping (1000 iterations) to obtain the 95% confidence interval (95%CI).

## 3. Results

The parents were on average 35 years old, and there were more mothers than fathers in the study population (Table 1). More mothers had asthma and hay fever compared to the fathers. The majority of the parents were never-smokers.

Mean air pollution exposures in the parents’ childhood were lowest in Umea and highest in Gothenburg (NO_2_ 14.0 and 38.0 µg/m_3_, PM_2.5_ 10.3 and 24.4 µg/m^3^, PM_10_ 16.5 and 28.6 µg/m_3_, BC 0.09 and 1.09 µg/m^3^), except for O_3,_ which was lowest in Bergen (62.7 µg/m^3^) and highest in Umea (68.4 µg/m^3^) (Table S12). Only annual mean values for PM_2.5_ and PM_10_ exceeded WHO recommendations in some centres (PM_2.5_ for parents 0–18 years old in Umea, Uppsala and Bergen; parents 0–18 years old and offspring 0–10 years old in Gothenburg; PM_10_ for parents 0–18 years old in Uppsala and Gothenburg). No annual mean exposures exceeded the recommended EU-values (Table S12).

The correlations between PM_2.5_, PM_10_, NO_2_ and BC were medium to strong, while O_3_ showed weaker correlation with the other pollutants (Table S10a,b). 

Maternal medium PM_2.5_ and PM_10_ exposure was associated with a higher risk of offspring early-onset asthma when compared to low exposure (Table 2). Maternal high PM_10_ exposure was associated with a higher risk of hay fever in offspring. Paternal medium O_3_ exposure increased the risk of offspring hay fever, while paternal high BC exposure reduced the risk of offspring early-onset asthma when compared to low exposure. NO_2_ and NDVI were not associated with any outcomes, neither in the maternal nor the paternal line.

Sensitivity analyses revealed protective associations of paternal high NDVI exposure (1000 m) for offspring early-onset asthma (Table S6b). Sensitivity analyses stratified by country and for parents born after 1985 gave roughly the same patterns, but with some variations due to low numbers (Tables S8a,b and S9a,b).

Maternal PM_10_ exposure had a direct effect on offspring early-onset asthma (Table 3) and an indirect effect on offspring hay fever (mediated by offspring’s own exposure and by exposure during pregnancy) (Table 3). Paternal O_3_ exposure was associated with increased odds for offspring hay fever through a direct and total effect, and was not mediated by O_3_ exposure during pregnancy.

## 4. Discussion

Exposure to PM_2.5_ and PM_10_ in mothers’ childhood was associated with higher risk of offspring early-onset asthma, and exposure to PM_10_ was associated with higher risk of offspring hay fever. Fathers’ exposure to O_3_ was associated with more offspring hay fever, while fathers’ BC exposure was associated with less offspring early-onset asthma. NO_2_ and NDVI were not significantly associated with any of offspring’s outcomes in neither the maternal nor paternal line, although a protective NDVI association was suggested with a larger buffer zone. The association between maternal exposure to PM_10_ and offspring early-onset asthma was a direct effect, while the effect on offspring hay fever was indirect, mediated by exposures during pregnancy and offspring’s own childhood. The association between paternal O_3_ exposure and offspring hay fever was direct and not mediated by other factors.

To the best of our knowledge, this is the first study to investigate the associations between individual exposures to air pollution and greenness during childhood of one generation on lung health and allergy in the second generation. In previous literature, the focus on the parents’ role in offspring’s health revolves around maternal factors and in particular exposures during pregnancy. Recent studies associating prenatal air pollution exposure in mothers with childhood asthma show how maternal environmental exposure just before and during pregnancy is critical for fetal lung development and future respiratory health [43,44,45]. Our results expand and elaborate on this, suggesting that also exposures as far back in time as the childhood of the parents may play an important role in offspring health.

A possible explanation for our findings is that potential epigenetic processes can be induced in response to environmental exposures and influence disease risk also in the next generation [46]. Even air pollution levels that are below recommended limit values may through such epigenetic processes have a potential harmful effect on the respiratory health of future offspring. While we found clearer signals in the maternal than in the paternal line, previous studies have identified associations between paternal exposures and offspring asthma. One study discovered an association between paternal smoking prior to conception and offspring non-allergic early-onset asthma, while other studies found associations between smoking and overweight onset in adolescent boys and increased risk of asthma in the next generation [25,27,47]. A similar pattern was observed in our study were fathers’ exposure to O_3_ was associated with higher risk of offspring hay fever. However, we also found a seemingly protective association between paternal BC exposure and offspring early-onset asthma. The estimates in the paternal line should be interpreted with caution due to low number of fathers in our analysis, but this is nevertheless a surprising result that should be investigated further. Ideally, information on both parents should be included in the same analyses to give a complete picture of the possible epigenetic processes. Unfortunately, we only had information on one parent and his/her offspring in our study, and not on entire family units. Analyses of offspring with both parents in a long timeframe should be emphasized in future research.

Our study revealed few associations between exposure to greenness and early-onset asthma or hay fever in offspring. This may be because we do not have data on the time spent in green spaces. In the sensitivity analyses performed for wider NDVI buffer zones, we observed protective associations for offspring early-onset asthma after parental exposure to high levels of NDVI. For offspring hay fever, NDVI exposure was on the contrary associated with increased risk. The latter is in line with existing literature, and is possibly due to pollen exposure triggering allergic disease [17,48].

A noteworthy feature in our study was that medium parental exposure levels were associated with significantly increased risk for offspring asthma and hay fever, despite the fact that these levels are quite low—even high exposure levels in our study were in fact well beyond the international recommended limit values. It appears that there were no clear dose–response relation between parental air pollution exposures and offspring disease risk—for offspring asthma, the risk was actually highest for those whose parents were medium exposed. This may be related to the importance of the exposure time window and the epigenetic processes discussed above. In a study by Svanes et al. [25], the age of smoking onset in the parents was an important risk factor for asthma in their offspring, even after adjustment for the number of cigarettes they had smoked before conception. Moreover, a recent epigenome-wide association study showed associations between pre-conception paternal smoking and DNA methylation characteristics in adult and adolescent offspring—independent of the amount smoked [49]. Our findings suggest that the same patterns may be present for air pollution exposures as for smoking exposures. Given the low levels of exposures, these results suggest the need for re-evaluation of the recommended limit values.

Associations between air pollutants are complex, and one could hypothesize that there are interactive effects at play. The focus of the present study on inter-generational effects of relatively low air pollution exposure is however still in its early days. There is a need to establish evidence that there are certain basic associations before moving on to disentangle whether these exposures depend on interactions and/or which pollutants are of most importance with regard to respiratory health in the next generation. The exploration of interactive inter-generational effects of air pollution components on lung health would be a valuable next step for future studies.

We focused on residential air pollution exposures, but exposures can be substantially higher when commuting, compared to being at home. Children spend only around 40–50% of their time at home [50]. However, in most Scandinavian cities, it is common to live in close proximity of the children’s school or kindergarten and it is therefore likely that the true everyday air pollution and greenness exposures are similar to the residential exposure levels.

Associations between maternal PM_10_ childhood exposure and offspring hay fever were mediated by offspring’s own exposure and by maternal pregnancy exposure, while the effect was direct and unmediated with regard to early-onset asthma in offspring. These findings may suggest that asthma risk is susceptible for an epigenetic transmission across generations, while risk for hay fever is more likely triggered by own exposures. This could in turn imply a different transmission susceptibility for allergic asthma and non-allergic asthma. Unfortunately, we could not distinguish between allergic and non-allergic asthma in our study.

Correlation analyses revealed a strong correlation between pregnancy exposure and offspring childhood exposure, but weaker correlation between parental childhood exposures and pregnancy/offspring childhood exposures. Many parents moved to other areas with other levels of exposures after they grew up, and then settled in the same area during pregnancy and upbringing of children. This was also illustrated by the mediation analyses, where the effects of maternal childhood exposures to pollutants on offspring hay fever were mediated in the same manner by exposure during pregnancy and offspring’s own childhood exposure.

There are several strengths of this study. The RHINESSA generation study was designed to study respiratory health across generations with detailed information on mothers and fathers and their offspring, making it possible to investigate different susceptibility time windows for developing disease. The detailed address history was collected for each participant, together with the standardized exposure assessment of numerous air pollutants. The extrapolation formulas from the LUR models enabled us to estimate concentrations for specific areas and time points by integrating data on topography, road network, traffic information and land use within geographic information systems, resulting in accurate exposure calculations also for unmonitored locations and years. Although we do not know the precise accuracy of our selected study centres, previous validation studies from the ESCAPE project have shown that the model has satisfactory accuracy, with 68 to 71% explained variance for the PM variables and 82% explained variance for NO2 [32,51].

Another strength is the mediation analysis to disentangle the effects of the exposures on the outcomes into the direct and indirect (mediated through offspring’s own exposure and maternal exposure during pregnancy) components, and the use of DAGs to avoid over-adjustment in our analyses and to identify the possible mediators.

Some limitations should be acknowledged. First, population-based studies are vulnerable to bias. The response rate in RHINESSA was fairly low (around 40%). However, compared to the general population in the same age range, the RHINESSA population did not differ substantially when looking at demographic distributions (e.g., sex, smoking habits, educational level and asthma status) [28]. Additionally, recall bias is a challenge in many population-based studies. However, we do not suspect this in our study—partly due to exposure data being objectively registered based on residential address histories from the Norwegian and Swedish population registries, and with air pollution exposures being modelled by land-use regression models and greenness exposures being assigned through satellite images. Furthermore, the outcomes (offspring asthma and offspring hay fever) were not dependent on the parents’ memory far back in time since their offspring were still young (mean age of 6 years). Second, in the current study, we tested numerous exposures for associations with the outcomes. This multiple testing can increase the possibility of more false positive findings due to type 1 error [52]. However, due to a relatively low sample size, we believe instead that there may be an under-estimation rather than over-estimation of the associations in our analyses. Thirdly, the use of back extrapolation methods in the air pollution assignments may be a weakness for assignments before 1990 since the 1990 estimates were used as a proxy for these years. Air pollutants (except for O_3_) had a large variation over time, which may cause error in the earliest years. However, in additional analyses where we excluded all parents born before 1985, the patterns remained roughly unchanged, with pollutants as asthma risk factors in the maternal line but not in the paternal line. Lastly, the information on included parents was self-reported through questionnaires, and information about children and grandparents was parent-reported, thus, posing a potential information bias. However, validation studies carried out in RHINESSA showed a minimal risk of bias for asthma, smoking status, body silhouettes and overweight status reported across generations [28,53,54].

## 5. Conclusions

In conclusion, this study found that air pollution exposure in a mother’s childhood appeared to be a risk factor for early-onset asthma and hay fever in her future offspring. The observed effect of maternal exposures on asthma was direct, while the effect on hay fever was partly mediated through both offspring’s own exposure and exposure during pregnancy. Results regarding fathers were inconclusive and should be investigated further. Furthermore, future research with larger study populations are needed to fully understand the intergenerational effects of air pollution and greenness on offspring asthma and hay fever. However, our results suggest that the current air pollution limit values may be too high and that the long-term effects of exposure to air pollution may have harmful effects even across generations.

## Figures and Tables

**Table 1:** Study population characteristics. *N* = 706 mothers and 400 fathers and their 1949 offspring.

Characteristics ^a^	RHINESSA	
	Fathers	Mothers
	*N* (%)	*N* (%)
N	400 (36.2)	706 (63.8)
Umea	88 (22.0)	166 (23.5)
Uppsala	85 (21.3)	136 (19.3)
Gothenburg	58 (14.5)	93 (13.1)
Bergen	169 (42.2)	311 (44.1)
Offspring sex (male)	327 (48.2)	630 (49.6)
Offspring mean age (SD)	5.4 (3.6)	6.1 (4.2)
Offspring early-onset asthma (<10 years of age)	60 (15.0)	141 (20.0)
Offspring hay fever	27 (6.8)	70 (9.9)
Parental mean age (SD)	35.0 (3.8)	34.6 (3.9)
Parental asthma	62 (15.5)	128 (18.1)
Early-onset asthma	31 (7.8)	35 (5.0)
Late-onset asthma	28 (7.0)	88 (12.5)
Parental hay fever	125 (31.3)	193 (27.3)
Parental smoking onset		
Never-smokers	271 (67.8)	427 (60.5)
Smokers before 18 years old	103 (25.8)	246 (34.8)
Smokers after 18 years old	26 (6.5)	31 (4.4)
Parental education		
Primary school	9 (2.3)	22 (3.1)
Secondary school	137 (34.3)	185 (26.2)
College/university	253 (63.3)	498 (70.5)
Grandparental asthma	45 (11.3)	74 (10.5)

Abbreviations: SD, standard deviation. ^a^ Missing information (N): parental early-onset asthma (13), parental late-onset asthma (13), parental hay fever (14), parental smoking onset (4), parental education (4), grandparental asthma (31).

**Table 2:** Univariable and multivariable analyses: associations between paternal (*N* = 400) and maternal (*N* = 706) exposure to air pollutants and NDVI and offspring (*N* = 1949) early-onset asthma (a) and hay fever (b) in the RHINESSA generation study.

**(a) Early-Onset Asthma.**									
		**Univariable**		**Multivariable ^3^**		**Univariable**		**Multivariable ^3^**	
**Exposure ^1^**	**Exposure Level ^2^**	**Fathers (OR, 95% CI)**	***p*^4^**	**Fathers (OR, 95% CI)**	***p*^4^**	**Mothers (OR, 95% CI)**	***p*^4^**	**Mothers (OR, 95% CI)**	***p*^4^**
**NO_2_**	Medium	1.15 (0.58–2.30)	0.690	1.09 (0.51–2.32)	0.824	**1.69 (1.05–2.73)**	**0.032**	1.78 (0.96–3.31)	0.067
	High	0.70 (0.34–1.44)	0.332	0.50 (0.21–1.20)	0.120	**1.68 (1.04–2.72)**	**0.034**	1.79 (0.89–3.60)	0.101
**PM_2.5_**	Medium	0.56 (0.27–1.14)	0.111	0.48 (0.20–1.14)	0.098	**2.09 (1.30–3.37)**	**0.002**	**2.23 (1.32–3.78)**	**0.003**
	High	0.70 (0.35–1.41)	0.320	0.53 (0.24–1.17)	0.115	1.55 (0.94–2.57)	0.088	1.66 (0.96–2.88)	0.072
**PM_10_**	Medium	0.49 (0.23–1.04)	0.064	0.46 (0.20–1.09)	0.077	**2.13 (1.35–3.38)**	**0.001**	**2.27 (1.36–3.80)**	**0.002**
	High	0.82 (0.42–1.62)	0.567	0.65 (0.31–1.40)	0.273	1.39 (0.83–2.31)	0.209	1.46 (0.84–2.53)	0.183
**BC**	Medium	1.26 (0.64–2.46)	0.501	0.86 (0.40–1.87)	0.707	1.60 (1.00–2.58)	0.051	1.45 (0.83–2.54)	0.186
	High	0.48 (0.22–1.04)	0.064	**0.31 (0.11–0.87)**	**0.026**	1.57 (0.98–2.53)	0.060	1.33 (0.69–2.58)	0.393
**O_3_**	Medium	1.90 (0.95–3.80)	0.071	1.93 (0.93–4.01)	0.079	0.81 (0.52–1.27)	0.366	0.86 (0.53–1.39)	0.542
	High	1.25 (0.60–2.60)	0.550	1.09 (0.42–2.82)	0.852	0.67 (0.42–1.06)	0.084	0.97 (0.52–1.82)	0.923
**NDVI (300 m)**	Medium	0.65 (0.30–1.42)	0.279	0.56 (0.26–1.20)	0.138	1.17 (0.74–1.85)	0.505	1.25 (0.79–2.00)	0.341
	High	0.76 (0.39–1.47)	0.411	0.67 (0.31–1.42)	0.297	0.78 (0.46–1.31)	0.341	1.00 (0.59–1.72)	0.987
**(b) Hay Fever.**									
		**Univariable**		**Multivariable ^3^**		**Univariable**		**Multivariable ^3^**	
**Exposure ^1^**	**Exposure Level ^2^**	**Fathers (OR, 95% CI)**	***p*^4^**	**Fathers (OR, 95% CI)**	***p*^4^**	**Mothers (OR, 95% CI)**	***p*^4^**	**Mothers (OR, 95% CI)**	***p*^4^**
**NO_2_**	Medium	1.67 (0.65–4.26)	0.285	2.72 (0.82–9.02)	0.103	1.13 (0.55–2.34)	0.740	1.52 (0.51–4.56)	0.454
	High	1.24 (0.45–3.40)	0.680	2.41 (0.60–9.65)	0.213	**2.01 (1.04–3.90)**	**0.039**	2.84 (0.88–9.19)	0.081
**PM_2.5_**	Medium	1.46 (0.48–4.45)	0.510	1.72 (0.44–6.80)	0.438	1.69 (0.83–3.46)	0.151	1.85 (0.85–4.00)	0.121
	High	2.26 (0.75–6.85)	0.149	2.78 (0.77–10.10)	0.120	1.97 (0.99–3.91)	0.052	1.90 (0.91–3.97)	0.086
**PM_10_**	Medium	1.24 (0.40–3.88)	0.708	1.90 (0.46–7.87)	0.375	1.71 (0.83–3.52)	0.147	1.85 (0.85–4.01)	0.121
	High	2.34 (0.78–7.00)	0.127	3.41 (0.87–13.30)	0.078	**2.44 (1.26–4.72)**	**0.008**	**2.66 (1.19–5.91)**	**0.017**
**BC**	Medium	2.10 (0.75–5.89)	0.160	2.52 (0.81–7.88)	0.112	1.50 (0.74–3.04)	0.257	1.70 (0.70–4.16)	0.243
	High	1.37 (0.46–4.05)	0.575	2.56 (0.70–9.37)	0.157	1.99 (1.00–3.97)	0.052	2.71 (0.96–7.65)	0.060
**O_3_**	Medium	**3.30 (1.16–9.40)**	**0.025**	**4.15 (1.28–13.50)**	**0.018**	1.33 (0.70–2.52)	0.383	1.56 (0.79–3.06)	0.198
	High	1.91 (0.63–5.80)	0.253	2.78 (0.58–13.26)	0.199	0.84 (0.42–1.68)	0.618	1.62 (0.54–4.82)	0.389
**NDVI (300 m)**	Medium	0.80 (0.27–2.36)	0.683	0.72 (0.24–2.14)	0.551	1.18 (0.60–2.33)	0.629	1.29 (0.65–2.57)	0.460
	High	1.22 (0.48–3.13)	0.681	1.35 (0.44–4.19)	0.602	1.15 (0.58–2.30)	0.683	1.57 (0.72–3.43)	0.257

Abbreviations: BC, black carbon; CI, confidence interval; NDVI, normalized difference vegetation index; NO_2_, nitrogen dioxide; O_3_, ozone; OR, odds ratio; PM_2.5_, particulate matter with an aerodynamic diameter lower than 2.5 µm; PM_10_, particulate matter with an aerodynamic diameter lower than 10 µm. ^1^ All air pollutants exposures were back-extrapolated in time with the ratio method. ^2^ The low exposure group was used as the reference group. ^3^ All models were adjusted for O_3_ and NDVI (300 m buffer), except for the O_3_ model which was adjusted for NO_2_ and NDVI (300 m buffer) and the NDVI model which was adjusted for O_3_ and NO_2_. All models were also adjusted for grandparental education and grandparental asthma. ^4^ All *p*-values < 0.05 = significant and marked bold.

**Table 3:** Mediation analysis of the association between parental exposure and offspring early-onset asthma and hay fever (outcome) through exposure during pregnancy and offspring own exposure (potential mediators).

**(a) Early-Onset Asthma.**				
**Mediator**	**Parental exposure**	**Offspring Early-onset Asthma**		
		**Total Effect**	**Indirect Effect**	**Direct Effect**
		OR (95% CI) *	OR (95% CI) *	OR (95% CI) *
Exposure during pregnancy (PM_10_)	**PM_10_ (maternal)**			
	Low	1.00	1.00	1.00
	Medium	**2.08 (1.31–3.31)**	1.10 (0.97–1.25)	**1.89 (1.17–3.06)**
	High	1.36 (0.85–2.19)	1.20 (0.96–1.50)	**1.13 (0.67–1.93)**
**(** **b) Hay Fever.**				
**Mediator**	**Parental exposure**	**Offspring Hay Fever**		
		**Total Effect**	**Indirect Effect**	**Direct Effect**
		OR (95% CI) *	OR (95% CI) *	OR (95% CI) *
Offspring own exposure (PM_10_)	**PM_10_ (maternal)**			
	Low	1.00	1.00	1.00
	Medium	1.75 (0.75–4.04)	**1.24 (1.08–1.44)**	1.40 (0.60–3.27)
	High	**2.70 (1.20–6.08)**	**1.73 (1.25–2.39)**	1.56 (0.66–3.69)
Exposure during pregnancy (PM_10_)	**PM_10_ (maternal)**			
	Low	1.00	1.00	1.00
	Medium	1.79 (0.79–4.08)	**1.49 (1.22–1.83)**	1.20 (0.52–2.74)
	High	**2.71 (1.24–5.93)**	**2.02 (1.49–2.76)**	1.34 (0.61–2.94)
Exposure during pregnancy (O_3_)	**O_3_ (paternal)**			
	Low	1.00	1.00	1.00
	Medium	**5.48 (1.50–20.1)**	1.10 (0.80–1.50)	**5.00 (1.31–19.1)**
	High	4.14 (0.69–24.9)	1.16 (0.70–1.94)	3.55 (0.53–24.0)

Abbreviations: CI, confidence interval; O_3_, ozone; OR, odds ratio; PM_10_, particulate matter with an aerodynamic diameter lower than 10 µm. * All *p*-values < 0.05 = significant and marked bold.

## Supplementary Materials

The following are available online at https://www.mdpi.com/1660-4601/17/16/5828/s1, Table S1. Overview of the models used to calculate air pollution exposures. Table S2. Landsat images used for NDVI calculations. Table S3. NDVI assignment to addresses. Table S4. Mean annual average exposure (range) for NDVI buffer zones per center for parental exposure (0–18 years) and offspring’s exposure (0–10 years). Table S5. Low, medium and high exposure categories for air pollutants for the time windows: parents (0–18 years) and offspring (0–10 years). Table S6. Associations of paternal (*N* = 400) and maternal (*N* = 706) exposure to additional buffer zones of NDVI with offspring (*N* = 1949) early-onset asthma (Table S6a) and hay fever (Table S6b) in the RHINESSA generation study. Table S7a–f. Correlation coefficients for the exposure time windows: parent (0–10 years) and parent (10–18 years) for each exposure. Table S8. Analyses stratified per country (Swedish centers versus Bergen): Associations between paternal (*N* = 400) and maternal (*N* = 706) exposure to air pollution and NDVI (300 m) and offspring (*N* = 1949) early-onset asthma (Table S8a) and hay fever (Table S8b) in the RHINESSA generation study. Table S9. Analyses for parents born after 1985: associations of paternal (*N* = 73) and maternal (*N* = 154) exposure to air pollutants and NDVI with offspring (*N* = 309) early-onset asthma (Table S9a) and hay fever (Table S9b) in the RHINESSA generation study. Table S10. Correlation coefficients for the included air pollutants and NDVI: parental exposure (S10a) and offspring exposure (S10b). Table S11a–f. Correlation coefficients for the exposure time windows: parent (0–18 years), pregnancy and offspring (0–10 years) per exposure. Table S12. Mean annual average exposure (range) for air pollutants and NDVI (300 m) per center for parent exposure (0–18 years) and offspring exposure (0–10 years). Figure S1. Directed acyclic graph for parental air pollution exposure and offspring’s early-onset asthma. Figure S2. Directed acyclic graph for parental greenness exposure and offspring’s early-onset asthma. Figure S3. Directed acyclic graph for parental air pollution exposure and offspring’s hay fever. Figure S4. Directed acyclic graph for parental greenness exposure and offspring’s hay fever.
