# Supplementary material for: Associations of Preconception Exposure to Air Pollution and Greenness with Offspring Asthma and Hay Fever
Source: Int J Environ Res Public Health. 2020 Aug 12;17(16):5828. doi: 10.3390/ijerph17165828 (PMC7459891; doi:10.3390/ijerph17165828)
Supplement: Supplementary file 1 [file ijerph-17-05828-s001.pdf]

Supplementary material

Table S1. Overview of the models used to calculate air pollution exposures.

Air pollutant	Study center	Model-year and source	
		2007	2010
**NO_2**	Umea, Uppsala, Gothenburg	-	de Hoogh et al 2016 (1)
	Bergen	-	de Hoogh et al 2016 (1)
**$PM_{2.5}$**	Umea, Uppsala, Gothenburg	-	de Hoogh et al 2016 (1)
	Bergen	-	de Hoogh et al 2016 (1)
**PM_{10}**	Umea, Uppsala, Gothenburg	Vienneau et al 2013 (2)	-
	Bergen	Vienneau et al 2013 (2)	-
**BC**	Umea, Uppsala, Gothenburg	-	de Hoogh et al 2018 (3)
	Bergen	-	de Hoogh et al 2018 (3)
**O_3**	Umea, Uppsala, Gothenburg	-	de Hoogh et al 2018 (3)
	Bergen	-	de Hoogh et al 2018 (3)

Abbreviations: BC, black carbon; NDVI, normalized difference vegetation index; NO_2, nitrogen dioxide; O_3, ozone; $PM_{2.5}$, particulate matter with an aerodynamic diameter lower than 2.5 µm; PM_{10}, particulate matter with an aerodynamic diameter lower than 10 µm.

Table S2. Landsat images used for NDVI calculations.

	**Bergen, 201/18**	**Gothenburg, 195/20**	**Gothenburg, 196/19**	**Umea, 193/15**	**Umea, 193/16**	**Uppsala, 193/18**	**Uppsala, 193/19**
**2014**	18/06/201, 8OLI	27/08/2014, 8OLI	21/08/2015, 8OLI	12/07/2014, 8OLI	25/07/2013, 8OLI	10/06/2014, 8OLI	10/06/2014, 8OLI
**2009**	03/07/2008, 5TM	26/06/2009, 5TM	01/06/2009, 5TM	28/06/2009, 5TM	28/06/2009, 5TM	28/06/2009, 5TM	28/06/2009, 5TM
**2004**	06/07/2003, 5TM	07/06/2002, 5TM	14/06/2002, 5TM	17/06/2005, 5TM	03/07/2005, 5TM	14/07/2003, 5TM	14/07/2003, 5TM
**1999**	03/06/1997, 5TM	17/06/2000, 5TM	08/06/2000, 5TM	20/07/1997, 5TM (194/15)	13/07/1997, 5TM	17/06/1999, 5TM	17/06/1999, 5TM
**1994**	29/07/1994, 5TM	30/06/1993, 5TM	24/06/1994, 5TM	05/07/1994, 5TM	05/07/1994, 5TM	05/07/1994, 5TM	05/07/1994, 5TM
**1989**	13/06/1989, 5TM	05/07/1989, 5TM	29/08/1989, 5TM	21/06/1989, 5TM	21/06/1989, 5TM	07/07/1989, 5TM	07/07/1989, 5TM
**1984**	18/06/1985, 5TM	27/06/1986, 5TM	02/06/1986, 5TM	26/06/1985, 5TM	26/06/1985, 5TM	09/07/1984, 5TM	09/07/1984, 5TM

Abbreviations: NDVI, normalized difference vegetation index; OLI, operational land imager; TM, thematic mapper.

Table S3. NDVI assignment to addresses.

**NDVI map**	**Address year**
1984	1975 to 1986

1989	1987 to 1991
1994	1992 to 1996
1999	1997 to 2001
2004	2002 to 2006
2009	2007 to 2011
2014	2012 to 2015

Abbreviations: NDVI, normalized difference vegetation index.

Table S4. Mean annual average exposure (range) for NDVI buffer zones per center for parental exposure (0-18 years) and offspring's exposure (0-10 years).

Average exposure (range)	Umea		Uppsala		Gothenburg		Bergen	
	Parent (0-18 years)	Offspring (0-10 years)	Parent (0-18 years)	Offspring (0-10 years)	Parent (0-18 years)	Offspring (0-10 years)	Parent (0-18 years)	Offspring (0-10 years)
**NDVI 100m**	0.565 (0.224-0.787)	0.514 (0.077-0.841)	0.581 (0.260-0.786)	0.574 (0.106-0.892)	0.532 (0.271-0.781)	0.608 (-0.056-0.862)	0.541 (0.097-0.747)	0.532 (0.107-0.771)
**NDVI 300m**	0.561 (0.276-0.777)	0.515 (0.154-0.815)	0.585 (0.376-0.768)	0.581 (0.216-0.846)	0.542 (0.236-0.710)	0.615 (0.170-0.833)	0.548 (0.188-0.773)	0.545 (0.096-0.788)
**NDVI 500m**	0.562 (0.296-0.780)	0.515 (0.169-0.823)	0.593 (0.391-0.758)	0.589 (0.212-0.871)	0.554 (0.211-0.723)	0.623 (0.150-0.838)	0.537 (0.157-0.751)	0.541 (0.116-0.762)
**NDVI 1000m**	0.564 (0.311-0.737)	0.518 (0.221-0.807)	0.611 (0.378-0.748)	0.599 (0.281-0.857)	0.561 (0.224-0.729)	0.620 (0.154-0.825)	0.527 (0.169-0.731)	0.523 (0.116-0.743)

Abbreviations: NDVI, normalized difference vegetation index.

Table S5. Low, medium and high exposure categories for air pollutants for the time windows: parents 0-18 years, offspring 0-10 years.

Range for exposure categories (based on tertiles)		Low	Medium	High	EU limit values	WHO guideline values
**NO_2**	**Parents 0-18 years**	<18.975	18.975-26.281	>26.281	40	40
	**Offspring 0-10 years**	<12.209	12.209-17.466	>17.466	40	40
**$PM_{2.5}$**	**Parents 0-18 years**	<13.655	13.655-16.859	>16.859	25	10
	**Offspring 0-10 years**	<8.208	8.208-10.102	>10.102	25	10
**PM_{10}**	**Parents 0-18 years**	<18.644	18.644-22.238	>22.238	40	20
	**Offspring 0-10 years**	<12.151	12.151-13.980	>13.980	40	20
**BC**	**Parents 0-18 years**	<0.443	0.443-0.903	>0.903	-	-
	**Offspring 0-10 years**	<0.297	0.297-0.558	>0.558	-	-
**O_3**	**Parents 0-18 years**	<63.700	63.700-67.144	>67.144	-	-
	**Offspring 0-10 years**	<64.289	64.289-67.823	>67.823	-	-
**NDVI (100m)**	**Parents 0-18 years**	<0.513	0.513-0.600	>0.600	-	-
	**Offspring 0-10 years**	<0.509	0.509-0.610	>0.610	-	-

**NDVI (300m)**	**Parents 0-18 years**	<0.520	0.520-0.597	>0.597	-	-
	**Offspring 0-10 years**	<0.522	0.522-0.612	>0.612	-	-
**NDVI (500m)**	**Parents 0-18 years**	<0.527	0.527-0.597	>0.597	-	-
	**Offspring 0-10 years**	<0.521	0.521-0.610	>0.610	-	-
**NDVI (1000m)**	**Parents 0-18 years**	<0.527	0.527-0.605	>0.605	-	-
	**Offspring 0-10 years**	<0.510	0.510-0.608	>0.608	-	-

Abbreviations: BC, black carbon; EU, European Union; NDVI, normalized difference vegetation index; NO_2, nitrogen dioxide; O_3, ozone; OR, odds ratio; $PM_{2.5}$, particulate matter with an aerodynamic diameter lower than 2.5 µm; PM_{10}, particulate matter with an aerodynamic diameter lower than 10 µm; WHO, World Health Organization.

Table S6. Associations of paternal (N = 400) and maternal (N = 706) exposure to additional buffer zones of NDVI with offspring (N = 1949) early onset asthma (table S8a) and hay fever (table S8b) in the RHINESSA generation study.

S6a. Early onset asthma

**Exposure**	**Exposure level**	**Univariable Fathers (OR, 95% CI)**	**p[2]**	**Multivariable[1] Fathers (OR, 95% CI)**	**p[2]**	**Univariable Mothers (OR, 95% CI)**	**p[2]**	**Multivariable[1] Mothers (OR, 95% CI)**	**p[2]**
**NDVI (100m)**	Medium	0.60 (0.28-1.27)	0.180	0.53 (0.26-1.08)	0.080	1.19 (0.75-1.87)	0.465	1.23 (0.78-1.96)	0.374
	High	0.76 (0.39-1.49)	0.421	0.69 (0.33-1.45)	0.325	0.76 (0.46-1.25)	0.273	0.94 (0.55-1.60)	0.820
**NDVI (500m)**	Medium	0.55 (0.26-1.20)	0.132	0.55 (0.24-1.25)	0.156	0.97 (0.62-1.52)	0.881	1.00 (0.63-1.59)	0.999
	High	0.67 (0.34-1.31)	0.238	0.62 (0.30-1.28)	0.194	0.61 (0.37-1.01)	0.055	0.75 (0.44-1.29)	0.303
**NDVI (1000m)**	Medium	**0.36 (0.15-0.83)**	**0.018**	**0.33 (0.14-0.79)**	**0.012**	0.97 (0.62-1.53)	0.904	1.05 (0.65-1.68)	0.844
	High	0.62 (0.32-1.18)	0.143	0.49 (0.23-1.07)	0.086	**0.59 (0.36-0.96)**	**0.034**	0.68 (0.41-1.15)	0.152

Abbreviations: CI, confidence interval; NDVI, normalized difference vegetation index; OR, odds ratio. [1] Performed for all significant results from the univariable analyses. All models were adjusted for O_3 and NO_2, and in addition adjusted for grandparental education and grandparental asthma.[2] All p-values < 0.05 = significant and marked bold.

S6b. Hay fever

**Exposure**	**Exposure level**	**Univariable Fathers (OR, 95% CI)**	**p[2]**	**Multivariable[1] Fathers (OR, 95% CI)**	**p[2]**	**Univariable Mothers (OR, 95% CI)**	**p[2]**	**Multivariable[1] Mothers (OR, 95% CI)**	**p[2]**
**NDVI (100m)**	Medium	0.76 (0.26-2.23)	0.620	0.69 (0.26-1.84)	0.460	1.66 (0.85-3.24)	0.141	1.92 (0.94-3.90)	0.072
	High	1.68 (0.62-4.58)	0.308	2.04 (0.62-6.73)	0.240	1.45 (0.72-2.91)	0.296	1.92 (0.83-4.46)	0.129
**NDVI (500m)**	Medium	0.88 (0.30-2.61)	0.818	0.87 (0.30-2.54)	0.799	1.75 (0.91-3.37)	0.096	1.89 (0.96-3.73)	0.065
	High	1.27 (0.50-3.26)	0.613	1.24 (0.43-3.62)	0.691	1.18 (0.57-2.47)	0.657	1.45 (0.63-3.34)	0.381
**NDVI (1000m)**	Medium	1.55 (0.51-4.72)	0.441	1.45 (0.50-4.23)	0.495	1.33 (0.66-2.66)	0.420	1.34 (0.66-2.72)	0.415
	High	2.08 (0.73-5.92)	0.170	1.80 (0.58-5.58)	0.310	1.39 (0.69-2.82)	0.360	1.69 (0.79-3.63)	0.180

Abbreviations: CI, confidence interval; NDVI, normalized difference vegetation index; OR, odds ratio. [1] Performed for all significant results from the univariable analyses. All models were adjusted for O_3 and NO_2, and in addition adjusted for grandparental education and grandparental asthma.[2] All p-values < 0.05 = significant and marked bold.

Table S7. Correlation coefficients for the exposure time windows: parent (0-10 years) and parent (10-18 years).

S7a. NO_2

	Parents (0-10 years)	Parents (10-18 years)
**Parents (0-10 years)**	1.0	0.876
**Parents (10-18 years)**	0.876	1.0

Abbreviations: NO_2, nitrogen dioxide.

S7b. $PM_{2.5}$

	Parents (0-10 years)	Parents (10-18 years)
**Parents (0-10 years)**	1.0	0.909
**Parents (10-18 years)**	0.909	1.0

Abbreviations: $PM_{2.5}$, particulate matter with an aerodynamic diameter lower than 2.5 µm.

S7c. PM_{10}

	Parents (0-10 years)	Parents (10-18 years)
**Parents (0-10 years)**	1.0	0.879
**Parents (10-18 years)**	0.879	1.0

Abbreviations: PM_{10}, particulate matter with an aerodynamic diameter lower than 10 µm.

S7d. BC

	Parents (0-10 years)	Parents (10-18 years)
**Parents (0-10 years)**	1.0	0.922
**Parents (10-18 years)**	0.922	1.0

Abbreviations: BC, black carbon.

S7e. O_3

	Parents (0-10 years)	Parents (10-18 years)

**Parents (0-10 years)**	1.0	0.903
**Parents (10-18 years)**	0.903	1.0

Abbreviations: O_3, ozone.

S7f. NDVI

	**Parents (0-10 years)**	**Parents (10-18 years)**
**Parents (0-10 years)**	1.0	0.728
**Parents (10-18 years)**	0.728	1.0

Abbreviations: NDVI, normalized difference vegetation index.

Table S8. Analyses stratified per country (Swedish centers versus Bergen): Associations between paternal (N = 400) and maternal (N = 706) exposure to air pollution and NDVI (300m) and offspring (N = 1949) early onset asthma (table S8a) and hay fever (table S8b) in the RHINESSA generation study.

S8a. Early onset asthma

**Exposure[1]**	**Centre**	**Exposure level**	**Univariable Fathers (OR, 95% CI)**	**p[3]**	**Multivariable[2] Fathers (OR, 95% CI)**	**p[3]**	**Univariable Mothers (OR, 95% CI)**	**p[3]**	**Multivariable[2] Mothers (OR, 95% CI)**	**p[3]**
**NO_2**	Swedish	Medium	2.14 (0.84-5.48)	0.113	1.77 (0.93-3.37)	0.081	1.53 (0.78-3.00)	0.213	**2.04 (1.07-3.87)**	**0.030**
		High	1.09 (0.44-2.75)	0.848	1.80 (0.80-4.05)	0.158	1.77 (0.94-3.36)	0.078	2.20 (1.00-4.84)	0.051
	Bergen	Medium	0.44 (0.17-1.18)	0.104	0.61 (0.15-2.41)	0.478	1.38 (0.69-2.78)	0.368	1.03 (0.43-2.47)	0.941
		High	**0.26 (0.08-0.86)**	**0.027**	0.43 (0.09-2.08)	0.295	1.28 (0.63-2.62)	0.497	0.91 (0.33-2.49)	0.857
**$PM_{2.5}$**	Swedish	Medium	1.12 (0.32-3.84)	0.862	1.19 (0.59-2.36)	0.629	1.36 (0.64-2.87)	0.422	1.39 (0.68-2.86)	0.368
		High	1.14 (0.46-2.84)	0.775	1.40 (0.70-2.82)	0.339	1.52 (0.81-2.85)	0.194	1.56 (0.77-3.15)	0.215
	Bergen	Medium	**0.28 (0.12-0.68)**	**0.005**	**0.23 (0.08-0.69)**	**0.009**	**2.45 (1.26-4.75)**	**0.008**	**2.69 (1.25-5.80)**	**0.011**
		High	-	-	-	-	1.76 (0.73-4.26)	0.207	2.13 (0.83-5.44)	0.115
**PM_{10}**	Swedish	Medium	0.65 (0.17-2.43)	0.524	1.02 (0.49-2.11)	0.954	1.46 (0.68-2.16)	0.330	1.26 (0.61-2.59)	0.536
		High	1.07 (0.43-2.62)	0.886	1.35 (0.72-2.52)	0.348	1.49 (0.81-2.74)	0.205	1.53 (0.82-2.87)	0.181
	Bergen	Medium	**0.33 (0.13-0.84)**	**0.020**	0.37 (0.13-1.07)	0.068	**2.22 (1.18-4.19)**	**0.014**	**2.31 (1.17-4.55)**	**0.016**
		High	0.56 (0.13-2.41)	0.433	0.80 (0.14-4.37)	0.792	1.04 (0.31-3.55)	0.948	0.98 (0.28-3.41)	0.974
**BC**	Swedish	Medium	1.21 (0.53-2.75)	0.644	1.23 (0.69-2.21)	0.483	1.46 (0.78-2.72)	0.233	1.27 (0.71-2.25)	0.421
		High	0.48 (0.16-1.45)	0.193	**0.34 (0.12-0.96)**	**0.043**	1.16 (0.58-2.30)	0.679	0.86 (0.38-1.98)	0.727
	Bergen	Medium	1.15 (0.28-4.67)	0.844	1.76 (0.41-7.59)	0.448	1.82 (0.70-4.75)	0.222	1.38 (0.49-3.92)	0.545
		High	0.42 (0.10-1.77)	0.236	0.94 (0.16-5.71)	0.950	1.95 (0.78-5-02)	0.166	1.51 (0.48-4.69)	0.480

**O_3**	Swedish	Medium	1.63 (0.41-6.41)	0.485	2.17 (0.60-7.86)	0.236	1.64 (0.59-4.53)	0.339	2.08 (0.74-5.86)	0.165
		High	1.09 (0.28-4.23)	0.901	2.30 (0.58-9.19)	0.237	1.31 (0.48-3.55)	0.600	2.16 (0.66-7.03)	0.201
	Bergen	Medium	2.21 (0.86-5.67)	0.098	1.81 (0.50-6.50)	0.362	0.72 (0.37-1.40)	0.337	0.75 (0.34-1.63)	0.467
		High	2.92 (0.52-16.4)	0.224	1.09 (0.09-12.6)	0.947	0.82 (0.30-2.24)	0.699	1.25 (0.33-4.64)	0.742
**NDVI (300m)**	Swedish	Medium	0.64 (0.22-1.84)	0.408	1.14 (0.64-2.05)	0.656	1.73 (0.86-3.48)	0.124	1.49 (0.84-2.64)	0.175
		High	0.54 (0.23-1.26)	0.153	0.93 (0.47-1.83)	0.841	1.02 (0.46-2.27)	0.966	1.08 (0.55-2.12)	0.814
	Bergen	Medium	0.68 (0.22-2.09)	0.507	0.63 (0.18-2.17)	0.463	0.81 (0.43-1.53)	0.516	0.80 (0.42-1.54)	0.509
		High	1.36 (0.47-3.93)	0.566	1.23 (0.43-3.49)	0.701	0.65 (0.33-1.28)	0.212	0.69 (0.32-1.49)	0.341

Abbreviations: BC, black carbon; CI, confidence interval; NDVI, normalized difference vegetation index; NO_2, nitrogen dioxide; O_3, ozone; OR, odds ratio; $PM_{2.5}$, particulate matter with an aerodynamic diameter lower than 2.5 µm; PM_{10}, particulate matter with an aerodynamic diameter lower than 10 µm. [1] All air pollutants exposures were back-extrapolated in time with the ratio method. [2] All models were adjusted for O_3 and NDVI (300m buffer), except for the O_3-model that was adjusted for NO_2 and NDVI (300m buffer) and the NDVI-model that was adjusted for O_3 and NO_2. All models were also adjusted for grandparental education and grandparental asthma. [3] All p-values < 0.05 = significant and marked bold. - = Too few observations.

S8b. Hay fever

**Exposure[1]**	**Centre**	**Exposure level**	**Univariable Fathers (OR, 95% CI)**	**p[3]**	**Multivariable[2] Fathers (OR, 95% CI)**	**p[3]**	**Univariable Mothers (OR, 95% CI)**	**p[3]**	**Multivariable[2] Mothers (OR, 95% CI)**	**p[3]**
**NO_2**	Swedish	Medium	**4.63 (1.34-16.01)**	**0.016**	**2.96 (1.28-6.89)**	**0.011**	2.24 (0.90-5.59)	0.083	**3.81 (1.60-9.14)**	**0.003**
		High	2.54 (0.72-9.02)	0.149	2.02 (0.57-7.11)	0.275	**3.10 (1.33-7.22)**	**0.009**	**3.80 (1.22-11.85)**	**0.021**
	Bergen	Medium	0.32 (0.07-1.43)	0.135	0.33 (0.04-3.05)	0.327	0.36 (0.11-1.21)	0.098	0.32 (0.05-2.21)	0.247
		High	0.18 (0.02-1.56)	0.119	0.52 (0.02-12.6)	0.688	0.80 (0.27-2.32)	0.676	0.76 (0.11-5.22)	0.778
**$PM_{2.5}$**	Swedish	Medium	1.95 (0.41-9.29)	0.402	1.05 (0.41-2.68)	0.915	1.15 (0.37-3.58)	0.804	1.32 (0.52-3.39)	0.561
		High	2.41 (0.62-9.41)	0.205	1.44 (0.58-3.54)	0.428	2.02 (0.93-4.39)	0.075	1.96 (0.84-4.60)	0.119
	Bergen	Medium	1.28 (0.26-6.23)	0.757	1.93 (0.32-11.6)	0.473	2.56 (0.84-7.85)	0.100	**3.02 (1.07-8.56)**	**0.037**
		High	-	-	-	-	0.51 (0.06-4.62)	0.553	-	-
**PM_{10}**	Swedish	Medium	1.34 (0.25-7.19)	0.736	1.04 (0.35-3.05)	0.947	1.31 (0.33-5.20)	0.699	1.46 (0.52-4.13)	0.471
		High	2.52 (0.66-9.66)	0.178	2.31 (0.94-5.69)	0.069	**2.83 (1.31-6.08)**	**0.008**	**2.94 (1.23-7.03)**	**0.016**
	Bergen	Medium	1.23 (0.25-6.11)	0.798	3.57 (0.33-39.1)	0.298	1.81 (0.64-5.07)	0.261	2.69 (0.83-8.77)	0.101
		High	-	-	-	-	-	-	-	-
**BC**	Swedish	Medium	2.38 (0.78-7.28)	0.128	1.87 (0.88-3.97)	0.104	**2.35 (1.05-5.26)**	**0.038**	**2.31 (1.08-4.96)**	**0.031**
		High	1.49 (0.42-5.29)	0.538	1.00 (0.28-3.53)	0.997	**3.00 (1.26-7.10)**	**0.013**	2.74 (0.95-7.93)	0.063
	Bergen	Medium	1.26 (0.32-4.98)	0.746	0.82 (0.21-3.18)	0.772	0.53 (0.12-2.24)	0.385	0.68 (0.11-4.35)	0.683
		High	-	-	-	-	0.94 (0.27-3.36)	0.919	1.15 (0.21-6.40)	0.874
**O_3**	Swedish	Medium	2.16 (0.46-10.26)	0.331	1.09 (0.28-4.24)	0.900	0.85 (0.34-2.14)	0.731	0.91 (0.34-2.42)	0.855

		High	1.03 (0.21-4.93)	0.975	0.86 (0.17-4.32)	0.853	0.54 (0.21-1.34)	0.181	0.97 (0.26-3.65)	0.961
	Bergen	Medium	3.06 (0.70-13.40)	0.139	1.95 (0.50-7.59)	0.334	1.28 (0.46-3.55)	0.635	1.09 (0.26-4.68)	0.904
		High	5.52 (0.50-60.90)	0.163	-	-	-	-	-	-
**NDVI (300m)**	Swedish	Medium	0.66 (0.18-2.38)	0.526	1.10 (0.52-2.35)	0.798	1.19 (0.53-2.68)	0.666	1.26 (0.62-2.56)	0.523
		High	0.76 (0.25-2.31)	0.623	1.46 (0.57-3.74)	0.425	0.76 (0.32-1.84)	0.545	1.36 (0.60-3.06)	0.462
	Bergen	Medium	2.16 (0.20-23.32)	0.527	1.95 (0.50-7.59)	0.334	1.13 (0.32-4.00)	0.851	1.00 (0.30-3.32)	0.997
		High	5.53 (0.63-48.49)	0.122	-	-	2.31 (0.75-7.14)	0.147	1.70 (0.44-6.66)	0.443

Abbreviations: BC, black carbon; CI, confidence interval; NDVI, normalized difference vegetation index; NO_2, nitrogen dioxide; O_3, ozone; OR, odds ratio; $PM_{2.5}$, particulate matter with an aerodynamic diameter lower than 2.5 µm; PM_{10}, particulate matter with an aerodynamic diameter lower than 10 µm. [1] All air pollutants exposures were back-extrapolated in time with the ratio method. [2] All models were adjusted for O_3 and NDVI (300m buffer), except for the O_3-model that was adjusted for NO_2 and NDVI (300m buffer) and the NDVI-model that was adjusted for O_3 and NO_2. All models were also adjusted for grandparental education and grandparental asthma. [3] All p-values < 0.05 = significant and marked bold. - = Too few observations.

Table S9. Analyses for parents born after 1985: Associations of paternal (N = 73) and maternal (N = 154) exposure to air pollutants and NDVI with offspring (N = 309) early onset asthma (Table 9a) and hay fever (Table 9b) in the RHINESSA generation study.

Table S9a. Early onset asthma

**Exposure[1]**	**Exposure level**	**Univariable Fathers (OR, 95% CI)**	**p[3]**	**Multivariable[2] Fathers (OR, 95% CI)**	**p[3]**	**Univariable Mothers (OR, 95% CI)**	**p[3]**	**Multivariable[2] Mothers (OR, 95% CI)**	**p[3]**
**NO_2**	Medium	1.21 (0.24-6.25)	0.816	0.39 (0.04-4.04)	0.430	2.40 (0.66-8.70)	0.183	7.76 (0.88-68.03)	0.064
	High	0.77 (0.14-4.42)	0.772	0.10 (0.00-3.09)	0.190	2.99 (0.85-10.47)	0.087	**14.0 (1.32-147.58)**	**0.028**
**$PM_{2.5}$**	Medium	-	-	-	-	2.22 (0.67-7.41)	0.195	2.65 (0.63-11.22)	0.184
	High	0.37 (0.05-2.99)	0.353	0.13 (0.01-3.27)	0.216	1.95 (0.51-7.49)	0.331	3.63 (0.85-15.48)	0.081
**PM_{10}**	Medium	0.18 (0.02-1.61)	0.126	**0.09 (0.01-0.88)**	**0.039**	1.61 (0.46-5.66)	0.456	1.92 (0.49-7.46)	0.349
	High	0.40 (0.05-3.28)	0.396	0.11 (0.00-4.77)	0.250	1.82 (0.49-6.80)	0.371	3.36 (0.89-12.71)	0.074
**BC**	Medium	1.83 (0.36-9.23)	0.462	1.15 (0.17-7.61)	0.885	3.06 (0.82-11.37)	0.095	4.84 (0.84-28.00)	0.078
	High	1.05 (0.16-6.66)	0.961	0.21 (0.01-4.23)	0.307	2.22 (0.59-8.30)	0.236	3.66 (0.44-30.84)	0.232
**O_3**	Medium	0.86 (0.16-4.56)	0.858	0.59 (0.06-5.48)	0.638	0.61 (0.14-2.59)	0.501	0.49 (0.11-2.07)	0.330
	High	0.33 (0.05-2.22)	0.256	0.08 (0.00-2.82)	0.163	0.61 (0.19-1.96)	0.407	3.79 (0.52-27.78)	0.190
**NDVI (300m)**	Medium	0.20 (0.02-1.73)	0.144	0.25 (0.03-2.32)	0.214	1.15 (0.31-4.22)	0.834	1.20 (0.29-5.01)	0.807
	High	0.59 (0.12-2.87)	0.511	0.41 (0.07-2.55)	0.340	0.25 (0.04-1.52)	0.132	0.26 (0.05-1.36)	0.111

Abbreviations: BC, black carbon; CI, confidence interval; NDVI, normalized difference vegetation index; NO_2, nitrogen dioxide; O_3, ozone; OR, odds ratio; $PM_{2.5}$, particulate matter with an aerodynamic diameter lower than 2.5 µm; PM_{10}, particulate matter with an aerodynamic diameter lower than 10 µm. [1] All air pollutants exposures were back-extrapolated in time with the ratio method. [2] All models were adjusted for O_3 and NDVI (300m buffer), except for the O_3-model that was adjusted for NO_2 and NDVI (300m buffer) and the NDVI-model that was adjusted for O_3 and NO_2. All models were also adjusted for grandparental education and grandparental asthma. [3] All p-values < 0.05 = significant and marked bold. - = Too few observations.

Table S9b. Hay fever

**Exposure**[1]	**Exposure level**	**Univariable Fathers (OR, 95% CI)**	**p**[3]	**Multivariable**[2] **Fathers (OR, 95% CI)**	**p**[3]	**Univariable Mothers (OR, 95% CI)**	**p**[3]	**Multivariable**[2] **Mothers (OR, 95% CI)**	**p**[3]
**NO_2**	Medium	-	-	-	-	2.14 (0.29-15.97)	0.458	5.78 (0.36-93.52)	0.216
	High	3.36 (0.19-59.54)	0.408	-	-	3.55 (0.56-22.46)	0.178	8.93 (0.32-245.97)	0.196
**$PM_{2.5}$**	Medium	-	-	-	-	3.67 (0.49-27.35)	0.205	4.68 (0.76-28.92)	0.097
	High	0.30 (0.02-5.56)	0.415	-	-	5.24 (0.83-33.13)	0.078	4.23 (0.54-33.17)	0.169
**PM_{10}**	Medium	-	-	-	-	3.62 (0.49-26.97)	0.209	3.80 (0.59-24.45)	0.160
	High	0.21 (0.01-3.85)	0.291	-	-	5.55 (0.88-35.14)	0.069	3.90 (0.60-25.41)	0.155
**BC**	Medium	-	-	-	-	2.28 (0.37-14.15)	0.376	3.11 (0.26-37.33)	0.371
	High	0.31 (0.02-5.48)	0.423	-	-	1.83 (0.24-13.91)	0.561	3.93 (0.17-90.61)	0.393
**O_3**	Medium	-	-	-	-	6.23 (0.71-54.25)	0.098	3.59 (0.36-35.86)	0.276
	High	1.94 (0.11-33.82)	0.648	-	-	2.54 (0.23-28.46)	0.450	6.57 (0.24-180.40)	0.266
**NDVI (300m)**	Medium	-	-	-	-	1.02 (0.14-7.38)	0.988	1.48 (0.14-15.50)	0.741
	High	0.38 (0.02-6.63)	0.506	-	-	1.11 (0.19-6.67)	0.906	1.84 (0.21-15.89)	0.580

Abbreviations: BC, black carbon; CI, confidence interval; NDVI, normalized difference vegetation index; NO_2, nitrogen dioxide; O_3, ozone; OR, odds ratio; $PM_{2.5}$, particulate matter with an aerodynamic diameter lower than 2.5 µm; PM_{10}, particulate matter with an aerodynamic diameter lower than 10 µm. [1] All air pollutants exposures were back-extrapolated in time with the ratio method. [2] All models were adjusted for O_3 and NDVI (300m buffer), except for the O_3-model that was adjusted for NO_2 and NDVI (300m buffer) and the NDVI-model that was adjusted for O_3 and NO_2. All models were also adjusted for grandparental education and grandparental asthma. [3] All p-values < 0.05 = significant and marked bold. - = Too few observations.

Table S10. Correlation coefficients for the included air pollutants and NDVI.

S10a. Parental exposure

**Air pollutant**	**$PM_{2.5}$**	**PM_{10}**	**NO_2**	**BC**	**O_3**	**NDVI**
**$PM_{2.5}$**	1.0	0.917	0.873	0.657	-0.404	-0.267
**PM_{10}**	0.917	1.0	0.793	0.639	-0.291	-0.251
**NO_2**	0.873	0.793	1.0	0.786	-0.651	-0.432
**BC**	0.657	0.629	0.786	1.0	-0.814	-0.310
**O_3**	-0.404	-0.291	-0.651	-0.814	1.0	0.380
**NDVI**[1]	-0.267	-0.251	-0.432	-0.310	0.380	1.0

Abbreviations: BC, black carbon; NDVI, normalized difference vegetation index; NO_2, nitrogen dioxide; O_3, ozone; $PM_{2.5}$, particulate matter with an aerodynamic diameter lower than 2.5 µm; PM_{10}, particulate matter with an aerodynamic diameter lower than 10 µm.
[1]300-m buffer.

S10b. Offspring's exposure

**Air pollutant**	**$PM_{2.5}$**	**PM_{10}**	**NO_2**	**BC**	**O_3**	**NDVI**

**$PM_{2.5}$**	1.0	0.966	0.742	0.697	-0.298	0.190
**PM_{10}**	0.966	1.0	0.725	0.738	-0.310	0.213
**NO_2**	0.742	0.725	1.0	0.872	-0.318	0.013
**BC**	0.697	0.738	0.872	1.0	-0.230	0.112
**O_3**	-0.298	-0.310	-0.318	-0.230	1.0	0.007
**NDVI[1]**	0.190	0.213	0.013	0.112	0.007	1.0

Abbreviations: BC, black carbon; NDVI, normalized difference vegetation index; NO_2, nitrogen dioxide; O_3, ozone; $PM_{2.5}$, particulate matter with an aerodynamic diameter lower than 2.5 µm; PM_{10}, particulate matter with an aerodynamic diameter lower than 10 µm.
[1]300-m buffer.

Table S11. Correlation coefficients for the exposure time windows: parent (0-18 years), pregnancy and offspring (0-10 years).

S11a. NO_2

	Parents (0-18 years)	Pregnancy	Offspring (0-10 years)
**Parents (0-18 years)**	1.0	0.418	0.433
**Pregnancy**	0.418	1.0	0.859
**Offspring (0-10 years)**	0.433	0.859	1.0

Abbreviations: NO_2, nitrogen dioxide.

S11b. $PM_{2.5}$

	Parents (0-18 years)	Pregnancy	Offspring (0-10 years)
**Parents (0-18 years)**	1.0	0.542	0.590
**Pregnancy**	0.542	1.0	0.839
**Offspring (0-10 years)**	0.590	0.839	1.0

Abbreviations: $PM_{2.5}$, particulate matter with an aerodynamic diameter lower than 2.5 µm.

S11c. PM_{10}

	Parents (0-18 years)	Pregnancy	Offspring (0-10 years)
**Parents (0-18 years)**	1.0	0.522	0.574
**Pregnancy**	0.522	1.0	0.801
**Offspring (0-10 years)**	0.574	0.801	1.0

Abbreviations: PM_{10}, particulate matter with an aerodynamic diameter lower than 10 µm.

S11d. BC

	Parents (0-18 years)	Pregnancy	Offspring (0-10 years)
**Parents (0-18 years)**	1.0	0.467	0.476
**Pregnancy**	0.467	1.0	0.857
**Offspring (0-10 years)**	0.476	0.857	1.0

Abbreviations: BC, black carbon.

S11e. O_3

	Parents (0-18 years)	Pregnancy	Offspring (0-10 years)
**Parents (0-18 years)**	1.0	0.465	0.478
**Pregnancy**	0.465	1.0	0.841
**Offspring (0-10 years)**	0.478	0.841	1.0

Abbreviations: O_3, ozone.

S11f. NDVI

	Parents (0-18 years)	Pregnancy	Offspring (0-10 years)
**Parents (0-18 years)**	1.0	0.205	0.176
**Pregnancy**	0.205	1.0	0.737
**Offspring (0-10 years)**	0.176	0.737	1.0

Abbreviations: NDVI, normalized difference vegetation index.

Table S12. Mean annual average exposure (range) for air pollutants and NDVI (300m) per center for parent exposure (0-18 years) and offspring exposure (0-10 years).

**Average exposure (range)[a]**	**Umea**		**Uppsala**		**Gothenburg**		**Bergen**		**EU limit values**	**WHO limit values**
	**Parent (0-18 years)**	**Offspring (0-10 years)**	**Parent (0-18 years)**	**Offspring (0-10 years)**	**Parent (0-18 years)**	**Offspring (0-10 years)**	**Parent (0-18 years)**	**Offspring (0-10 years)**		
**NDVI 300m**	0.561 (0.276-0.777)	0.515 (0.154-0.815)	0.585 (0.376-0.768)	0.581 (0.216-0.846)	0.542 (0.236-0.710)	0.615 (0.170-0.833)	0.548 (0.188-0.773)	0.545 (0.096-0.788)		
**NO_2 µg/m^3**	14.0 (1.3-33.4)	10.9 (0.3-34.1)	22.5 (5.4-46.3)	14.4 (2.6-33.5)	38.0 (15.3-69.7)	19.0 (2.3-40.5)	23.7 (2.9-44.9)	16.1 (3.4-33.4)	40[b]	40[b]
**$PM_{2.5}$ µg/m^3**	10.3 (1.2-20.0)	7.3 (0.5-19.1)	17.4 (9.8-25.7)	9.9 (4.5-17.6)	24.4 (14.8-29.8)	11.9 (6.0-17.1)	14.5 (3.9-22.8)	8.9 (2.4-14.9)	25[b]	10[b]
**PM_{10} µg/m^3**	16.5 (11.8-25.1)	11.3 (7.6-19.3)	23.7 (16.7-32.5)	14.2 (9.2-20.2)	28.6 (19.8-37.0)	15.0 (10.8-20.9)	19.7 (13.3-27.0)	13.0 (7.7-18.9)	40[b]	20[b]
**BC µg/m^3**	0.09 (0-1.09)	0.23 (0-1.50)	0.64 (0.20-1.42)	0.52 (0-1.45)	1.09 (0.51-1.89)	0.67 (0.10-1.59)	0.91 (0-2.43)	0.45 (0-1.21)	-	-

**O_3 µg/m³**	68.4 (62.6-73.3)	67.6 (58.1-75.0)	67.4 (62.3-71.3)	68.4 (56.3-75.5)	64.2 (57.6-70.5)	67.1 (57.3-76.2)	62.7 (51.2-74.6)	64.0 (54.3-76.6)	-[c]	-[c]

Abbreviations: BC, black carbon; EU, European Union; NDVI, normalized difference vegetation index; NO_2, nitrogen dioxide; O_3, ozone; $PM_{2.5}$, particulate matter with an aerodynamic diameter lower than 2.5 µm; PM_{10}, particulate matter with an aerodynamic diameter lower than 10 µm; WHO, World Health Organization.[a] All air pollutants exposures were back-extrapolated in time with the ratio method. [b] Annual mean values. [c] Only maximum daily 8-hour mean values available.

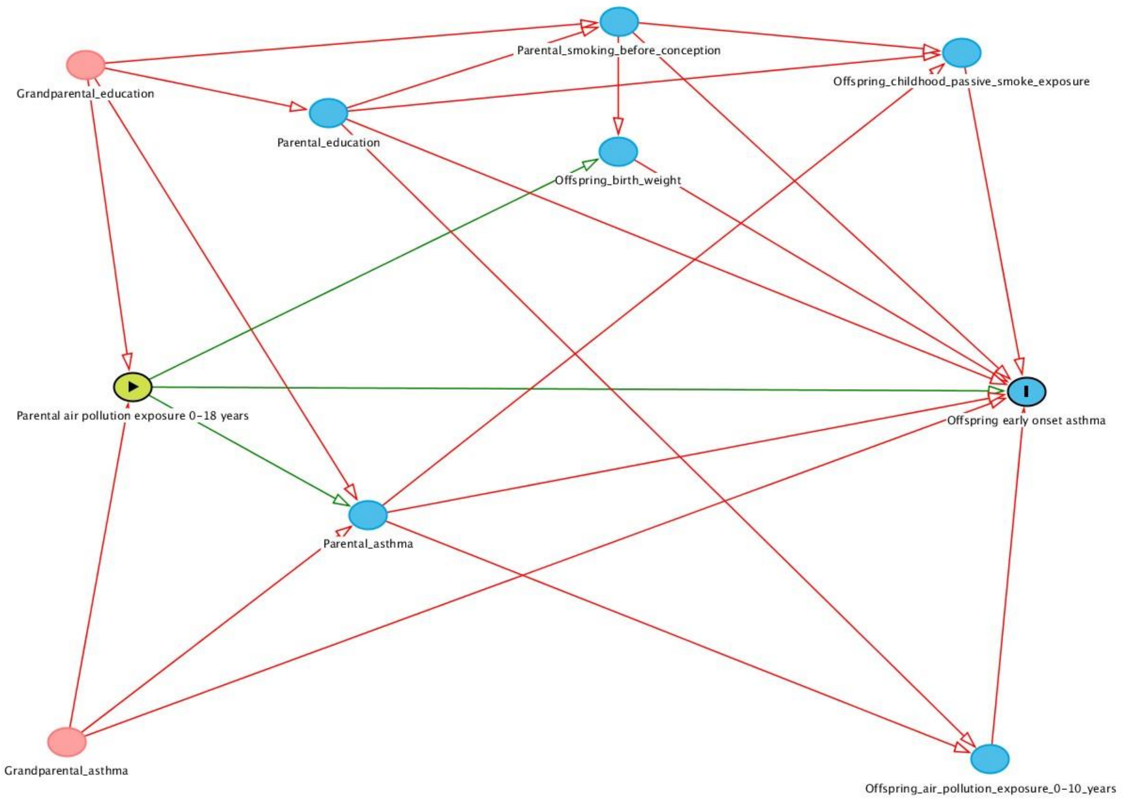

Figure S1. Directed Acyclic Graph for parental air pollution exposure and offspring's early onset asthma. Green circle with arrow: main exposure in the analysis. Blue circle with "I": main outcome. Other blue circles: risk factors for the outcome that are not risk factors for the exposure. Red circles: risk factors for both the outcome and the main exposure. Green arrows: paths from the main exposure. Red arrows: paths from other risk factors.

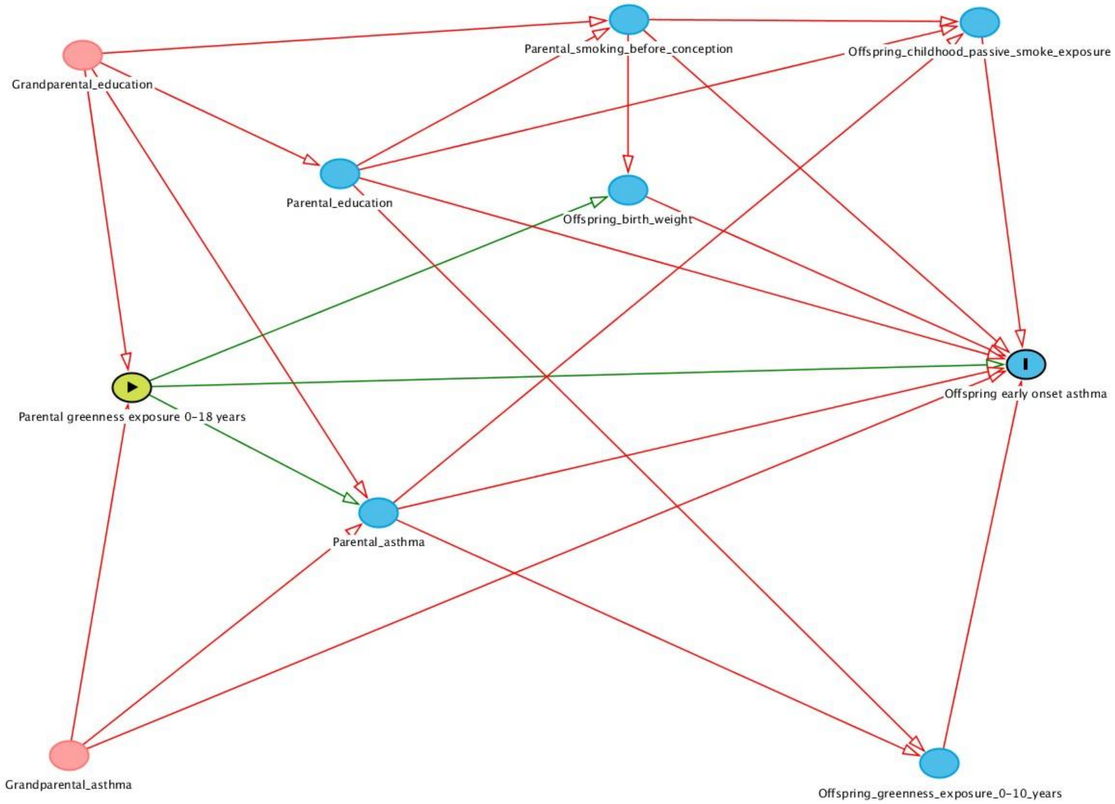

Figure S2. Directed Acyclic Graph for parental greenness exposure and offspring's early onset asthma. Green circle with arrow: main exposure in the analysis. Blue circle with "I": main outcome. Other blue circles: risk factors for the outcome that are not risk factors for the exposure. Red circles: risk factors for both the outcome and the main exposure. Green arrows: paths from the main exposure. Red arrows: paths from other risk factors.

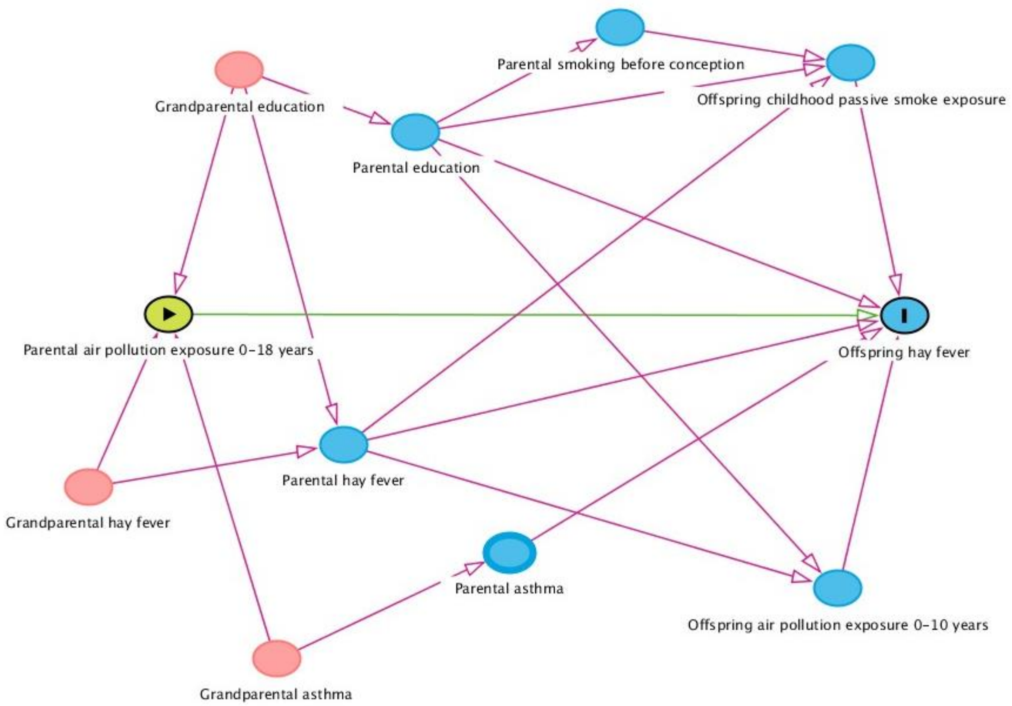

Figure S3. Directed Acyclic Graph for parental air pollution exposure and offspring's hay fever.
Green circle with arrow: main exposure in the analysis. Blue circle with "I": main outcome. Other blue circles: risk factors for the outcome that are not risk factors for the exposure. Red circles: risk factors for both the outcome and the main exposure. Green arrows: paths from the main exposure. Red arrows: paths from other risk factors.

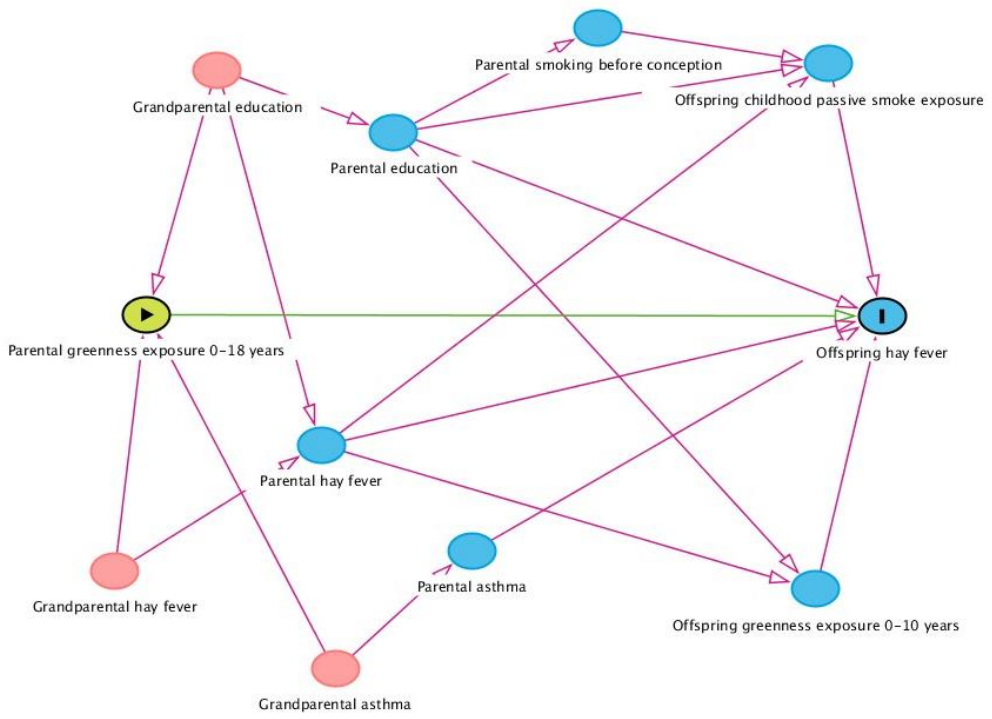

Figure S4. Directed Acyclic Graph for parental greenness exposure and offspring's hay fever. Green circle with arrow: main exposure in the analysis. Blue circle with "I": main outcome. Other blue circles: risk factors for the outcome that are not risk factors for the exposure. Red circles: risk factors for both the outcome and the main exposure. Green arrows: paths from the main exposure. Red arrows: paths from other risk factors.

References

1. de Hoogh K, Gulliver J, Donkelaar AV, Martin RV, Marshall JD, Bechle MJ, et al. Development of West-European PM2.5 and NO2 land use regression models incorporating satellite-derived and chemical transport modelling data. Environmental research. 2016;151:1-10 DOI: 10.1016/j.envres.2016.07.005.

2. Vienneau D, de Hoogh K, Bechle MJ, Beelen R, van Donkelaar A, Martin RV, et al. Western European land use regression incorporating satellite- and ground-based measurements of NO2 and PM10. Environmental science & technology. 2013;47(23):13555-64 DOI: 10.1021/es403089q.

3. de Hoogh K, Chen J, Gulliver J, Hoffmann B, Hertel O, Ketzel M, et al. Spatial PM2.5, NO2, O3 and BC models for Western Europe - Evaluation of spatiotemporal stability. Environment international. 2018;120:81-92 DOI: 10.1016/j.envint.2018.07.036.
